# Echocardiographic Evaluation of Aortic Stenosis: A Comprehensive Review

**DOI:** 10.3390/diagnostics13152527

**Published:** 2023-07-29

**Authors:** Rachele Manzo, Federica Ilardi, Dalila Nappa, Andrea Mariani, Domenico Angellotti, Maddalena Immobile Molaro, Giulia Sgherzi, Domenico Simone Castiello, Fiorenzo Simonetti, Ciro Santoro, Mario Enrico Canonico, Marisa Avvedimento, Raffaele Piccolo, Anna Franzone, Giovanni Esposito

**Affiliations:** Department of Advanced Biomedical Sciences, Division of Cardiology, Federico II University, 80131 Naples, Italy

**Keywords:** aortic stenosis, echocardiography, aortic stenosis grading, artificial intelligence, multiparametric approach, cardiac damage

## Abstract

Echocardiography represents the most important diagnostic tool in the evaluation of aortic stenosis. The echocardiographic assessment of its severity should always be performed through a standardized and stepwise approach in order to achieve a comprehensive evaluation. The latest technical innovations in the field of echocardiography have improved diagnostic accuracy, guaranteeing a better and more detailed evaluation of aortic valve anatomy. An early diagnosis is of utmost importance since it shortens treatment delays and improves patient outcomes. Echocardiography plays a key role also in the evaluation of all the structural changes related to aortic stenosis. Detailed evaluation of subtle and subclinical changes in left ventricle function has a prognostic significance: scientific efforts have been addressed to identify the most accurate global longitudinal strain cut-off value able to predict adverse outcomes. Moreover, in recent years the role of artificial intelligence is increasingly emerging as a promising tool able to assist cardiologists in aortic stenosis screening and diagnosis, especially by reducing the rate of aortic stenosis misdiagnosis.

## 1. Introduction

**Figure 1:**
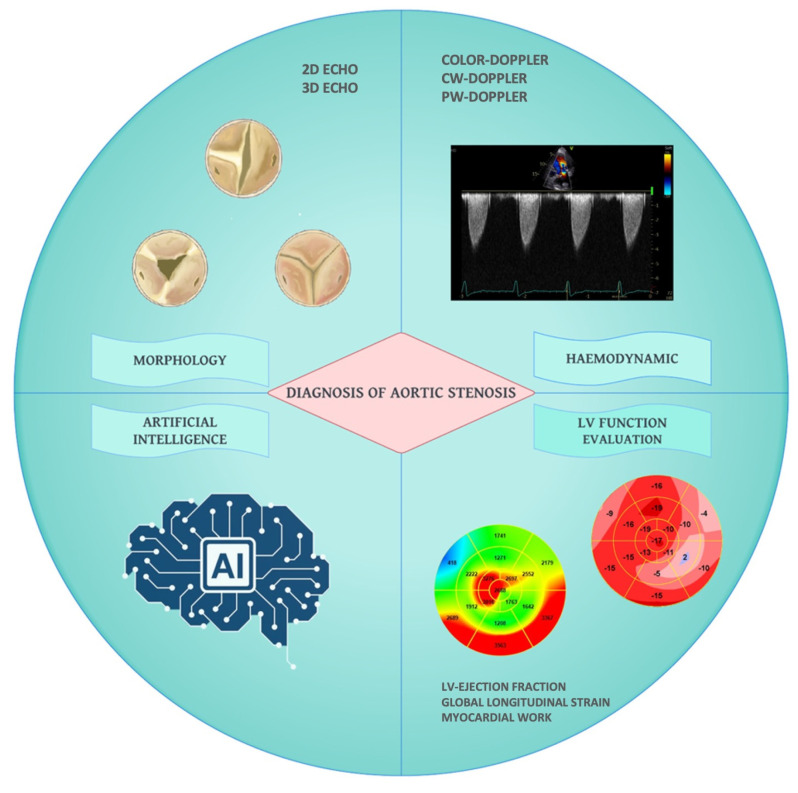
Comprehensive echocardiographic evaluation of aortic stenosis. CW: continuous wave; LV: left ventricle; PW: pulsed wave; 2D: two-dimensional; 3D: three-dimensional.

Aortic stenosis (AS) is the most frequent valvular heart disease (VHD) worldwide, expected to increase in prevalence due to the aging population. Statistical modeling has estimated an incidence rate of severe AS ranging from 4% to 7% per year among people ≥65 years of age [1]. Echocardiography represents the most important diagnostic tool in the identification of this VHD playing a crucial role in the periprocedural phase and during the long-term follow-up of patients undergoing surgical or transcatheter aortic valve replacement (AVR) [2]. In recent years, several innovations in the field of echocardiography have improved the accuracy in the diagnosis of AS, as well as in the identification of the related cardiac structural changes which have prognostic implications. This review aims to provide an overview of the latest technological advancements in the echocardiographic assessment of AS and their routine use in the clinical practice (Figure 1).

## 2. Aortic Stenosis Etiology and Echocardiographic Morphologic Assessment

The first echocardiographic approach in the evaluation of AS begins with the characterization of aortic valve morphology by transthoracic parasternal long- and short-axis views, in order to identify the number, mobility, thickness, and calcification of the cusps and to evaluate the etiology [3].

Two-dimensional (2D) and M-mode techniques are quick and relatively easy ways to assess cusps mobility: the first method allows the direct visualization of the reduced systolic cusps excursion, while M-mode provides a rough estimate of the severity of AS by identifying the maximal degree of separation of the leaflets during systole, with a maximal opening of at least 1.5 cm virtually excluding significant AS.

**Figure 2:**
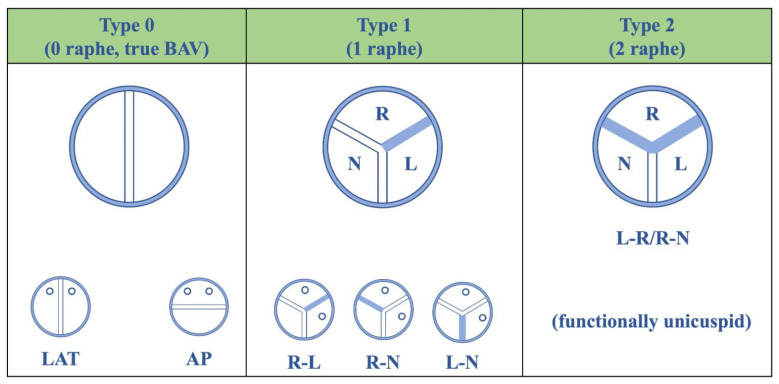
Classification system for bicuspid aortic valves adapted by Sievers et al. AP: Anterior-Posterior; BAV: Bicuspid aortic valve; L: Left coronary sinus; LAT: Lateral; N: Non-coronary sinus; R: Right coronary sinus.

Calcific degeneration, by far, is the most common cause of aortic valve stenosis [4] and has an estimated prevalence of 0.4% in the general population and 1.7% in the population >65 years of age, in developed countries [5]. Degenerative calcific AS is an active process characterized by the disruption of the aortic valvular endothelium with endothelial dysfunction, lipid accumulation, and infiltration of lymphocytes and macrophages that release pro-inflammatory molecules; they recruit fibroblasts and activate osteoblasts, leading to valve fibrosis, progressive thickening, that, over time, evolves into severe valve calcification [6]. Therefore, valve calcification leads to restriction of leaflets mobility, valve area reduction, and flow obstruction. Despite the presence of important calcification that could make the detection of the underlying valve anatomy very challenging, some aspects might be helpful in the diagnosis. Firstly, the prevalence of tricuspid or bicuspid valves, as the underlying anatomy of calcific AS, relies on age [7], as bicuspid valves are much more frequent in younger patients. The prevalence of bicuspid aortic valve ranges between 0.5% and 1% [8,9] and different fusion patterns are described: in about 75% of cases, the fusion is between the right and the left coronary cusps, in about 14% of cases is between the right and non-coronary cusps, while the fusion between the left and non-coronary cusps or a bicuspid valve with two same-size cusps are less-common findings (Figure 2).

In addition, bicuspid aortic valves frequently show an asymmetric closure line and diastolic doming in the long-axis view, together with an elliptical orifice during systole in the short-axis view. The localization of calcification may be peculiar: tricuspid valves usually develop calcification more prominent in the central and basal part of each cusp, while bicuspid valves often have an asymmetric pattern of calcification, related to the type of cuspid fusion [3]. Conversely, aortic valve calcification located along the edges of the cusps leading to commissural fusion, with a characteristic triangular systolic orifice, is suggestive of rheumatic heart disease (RHD) as the etiologic cause of AS. Although the prevalence of rheumatic disease, which is an immune-mediated multisystem inflammatory disease caused by an untreated throat infection with group A Streptococcus, is decreasing in industrialized countries, about 34 million people worldwide are affected by RHD, resulting in 340,000 deaths and 10 million disability-adjusted life-years lost per year [10].

The diagnosis of congenital AS is rare in adult patients as this defect is usually discovered early in the neonatal or young ages [3]. Other less common anatomical variants are the unicuspid aortic valve, which can be associated with AS even without heavy calcification and has a prevalence of about 0.02% [11], and the quadricuspid aortic valve, frequently associated with aortic regurgitation [12].

Visual assessment of valve morphology and leaflet movement should be an essential part of the comprehensive evaluation of AS. The degree of calcification, estimated by the presence of increased echogenicity and thickening of the leaflets, can provide prognostic relevance for predicting the need for later aortic valve replacement and mortality. Nemchyna et al., in their study, developed the “visual score”, a new tool to standardize, as much as possible, the morphologic assessment of the aortic valve. This semi-quantitative score is obtained from the sum of the points given to some different parameters (thickening of leaflets, echogenicity, localization of lesions, and mobility of leaflets); it ranges from a minimum of 0 to a maximum of 11 points, with a value of 9 for women and of 10 for men able to predict severe AS with high specificity. This tool has demonstrated a good correlation with aortic valve hemodynamic parameters and with the calcium score obtained by multi-slice computed tomography (MSCT) [13].

Furthermore, in order to better understand the nature and the localization of the stenosis, the Color Doppler and the pulsed-wave (PW) Doppler methods can precisely define the site of increased velocity and, together with the anatomical evaluation of the left ventricle outflow tract (LVOT) and the ascending aorta, they allow to discriminate between valvular, supra- or sub-valvular obstructions. 

**Figure 3:**
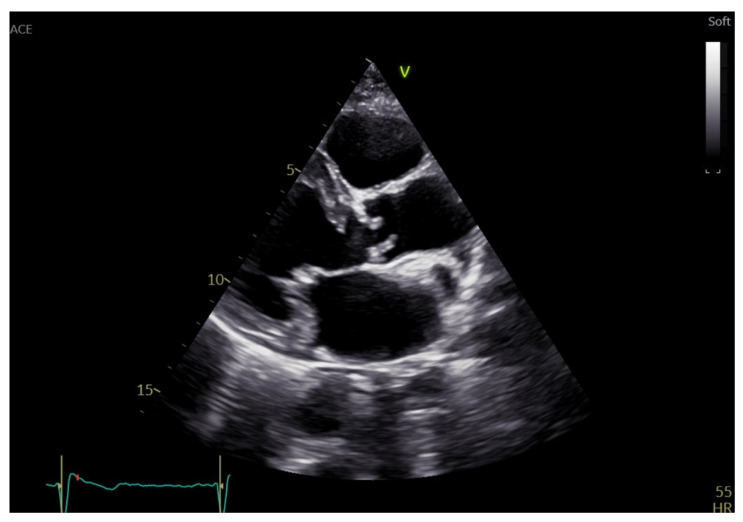
Subaortic discrete membrane.

Sub-valvular AS can be fixed or dynamic: the first one can be accounted for many anatomical features, including a discrete fibrous membrane, a thick fibromuscular ridge, or a tubular fibromuscular channel along the LVOT [14] (Figure 3). Dynamic obstruction, often linked to hypertrophic cardiomyopathy, refers to a form that changes in severity during the cardiac cycle and prevails during mid-to-late systole with a late peak in the velocity curve [3]. Supra-valvular AS is a rare condition caused by systemic elastin arteriopathy that can be associated or not with syndromic conditions, such as William’s syndrome [15]. The most frequently described variant is the so-called “hourglass”, given by pronounced aortic thickening with a resulting annular ridge at the superior margin of the Valsalva sinuses [16].

## 3. Echocardiographic Assessment of AS Severity

The first hemodynamic parameter that needs to be evaluated when searching for AS is the aortic jet velocity (Table 1) measured with continuous-wave (CW) Doppler [17]. 

Careful attention must be given to the transducer position, in order to obtain a parallel alignment between the aortic jet and the ultrasound beam. Sometimes, a small dual-crystal CW transducer (pencil or Pulse Echo Doppler Flow Velocity meter probe [PEDOF]) is recommended to achieve optimal angulation [3]. The maximum velocity (Vmax) will be obtained from the spectral Doppler signal, in correspondence with the outer edge at the peak of the curve, paying attention to not include the fine linear signals due to transit-time effects. The shape of the CW waveform can help determine the severity of the AS, with an early peak in moderate AS and slow acceleration with a late peak in severe AS. CW waveform is also helpful in identifying the dynamic nature of the obstruction since subaortic dynamic obstruction shows a characteristic late-peaking velocity curve, usually concave upward in early systole. At least three beats should be analyzed for patients in sinus rhythm and five beats in case of irregular rhythms. Post-extrasystolic beats should always be excluded from the measurement [3]. Multiple acoustic windows should always be used to determine the highest velocity [3]. Thaden et al. reported that in a contemporary cohort of 100 patients with AS, Vmax was found outside the apical imaging window in 61% of patients, most commonly in the right parasternal window (50%) [18]. They proved that neglecting non-apical windows results in the misclassification of AS in up to 23% of patients [18]. Benfari et al. showed that the right parasternal view (RPV) is a feasible approach in the vast majority of patients with AS, with a reclassification of severity towards moderate or severe AS in up to 13–26% of patients [19]. Moreover, a faster rate of AS progression has been described when using the right parasternal view, as well as a reduction in the inconsistencies between mean gradient and aortic valve area (AVA) [19]. In addition, searching for the highest velocity is of crucial importance since it has a prognostic significance: several studies evaluated the relationship between Vmax and long-term clinical outcomes in patients with severe AS [20,21,22,23]. Nakatsuma et al. in their study demonstrated that in a population of conservatively managed severe AS patients, increasing Vmax was associated with an incrementally higher risk for aortic valve-related death or heart failure hospitalization [24]. There was no significant interaction between the symptomatic status and the effect of Vmax on AS-related events, supporting the guidelines recommendation on AVR in asymptomatic patients with very severe AS (Vmax ≥ 5.0 m/s, Class IIa Level B of recommendation) [24,25]. Also, a Vmax progression ≥ 0.3 m/s/year has been retained as an indication for AVR in this patient population [25]. A recent meta-analysis of 24 prospective studies evaluated the most up-to-date mean annualized rates of AS progression determined by hemodynamic and anatomic indices. In particular, in a subset of moderate AS patients, there was a mean annualized peak velocity progression of 0.18 m/s/y [95% CI: 0.12–0.23 m/s/y]. Based on the upper 95% CI for progression of 0.23 m/s/y, the authors suggested that an even lower threshold for the definition of rapid progression than the one previously reported, may be used [6]. 

An essential parameter when assessing AS severity is the transvalvular aortic gradient (Table 1), the pressure difference between the left ventricle (LV) and the aorta during the entire systole. It can be derived from the simplified Bernoulli equation [3]. Since peak gradient derives from peak velocity, it does not add further information and thus is not considered in the stratification of AS severity. Furthermore, the peak gradient represents the maximum instantaneous pressure difference and not the difference between the peak LV pressure and the peak aortic pressure, which do not occur at the same time point. The Doppler peak gradient is expected to be always higher than the Cath-lab-derived one [3]. 

Conversely, the mean gradient, which represents the average gradient during the entire systole, has been shown to better correlate with the peak-to-peak gradient obtained during cardiac catheterization, and should always be measured. It is calculated electronically by tracing the CW spectral waveform [26].

When assessing pressure gradients across stenotic aortic valves, some important concerns have to be considered. At first, across the narrowed valve, the conversion of potential energy into kinetic energy results in a higher velocity and a pressure drop. Distal to the orifice, part of the kinetic energy is lost as heat due to turbulences and viscous losses, and part is reconverted into potential energy, leading to a pressure recovery (PR) in the ascending aorta. The entity of PR can be calculated as follows: *PR = 4v*^2^ × 2*EOA/AoA* × [1 (*EOA*/*AoA*)] [3]. This phenomenon tends to occur in the presence of a small ascending aorta: in this case, the gradual widening from the aortic valve towards the ascending tract is unfavorable for the development of turbulences. CW Doppler assesses the pressure drop from the LV to the vena contracta and does not take into account the recovery of pressure occurring distally in the ascending aorta. Since CW Doppler leads to an overestimation of the pressure gradient, the PR phenomenon should be considered in those patients with an ascending aorta diameter <30 mm [3]. 

Differently from flow velocity and pressure gradients that vary with cardiac output (Table 1), AVA should always be calculated, particularly in patients with very low or very high flow rates, since it has higher stability over a range of hemodynamic states [27]. AVA is derived from the continuity equation based on the conservation of mass. It assumes that the volume of blood flowing through the LVOT is the same as that of blood flowing through the aortic valve. Stroke volume (SV) is given by the product of the velocity–time integral (VTI) and the cross-sectional area (CSA) of the conduit so that AVA can be calculated as follows: *AVA* = (*CSA_LVOT_* × *VTI_LVOT_*)/*VTI_AV_* [26].

The continuity equation requires the measurement of AS jet velocity by CW Doppler, of LVOT diameter for calculation of the CSA, and of LVOT velocity by PW Doppler [3]

LVOT diameter should be systematically reported in each echocardiographic exam to allow accurate monitoring of stenosis severity progression during the follow-up. LVOT is obtained in the parasternal long-axis view, at the base of the aortic valve cusps or 1 to 5 mm below the aortic annulus, in mid-systole, using an inner-edge to inner-edge methodology. There is wide variability in LVOT measurements, ranging from 5% to 8%, thus, it represents the most significant source of error in the continuity equation. Some studies suggest that the evaluation of the LVOT diameter at a distance of >5 to 10 mm below the aortic annulus (proximal LVOT diameter, relative to the direction of flow) is less accurate and reproducible for the estimation of stroke volume and AVA [28]. The recently published World Alliance of Societies of Echocardiography (WASE) Study for Normative Values of AVA and aortic valve Doppler measurements showed statistically significant sex-related differences in LVOT diameter, with low normal limit as small as 1.6 cm in older women, and upper normal limit as large as 2.6 in young men; no major differences were found between ages groups [29].

LVOT velocity is recorded using PW Doppler in a five-chamber view or in the apical long-axis view, just below the aortic valve. Optimal PW spectral waveform should have a narrow band of recorded velocities throughout the systole. Subaortic dynamic obstruction with high subaortic velocities, as well as concomitant aortic regurgitation, with high subaortic flow rates, may limit the accuracy of SV measurements [3].

AVA can also be obtained with the planimetry method, by transthoracic echocardiography (TTE) or TEE, by directly tracing the anatomical orifice of the aortic valve. 

The WASE study also showed statistically significant differences in AVA between sexes, in all age groups, persisting even after BSA indexing [29]. Although the guidelines proposed an AVAi cut-off value of 0.6 cm^2^/m^2^ for severity, due to the differences attributable to BSA, a new cut-off value of 0.5 cm^2^/m^2^ has been proposed. This value is able to identify a subgroup of patients with higher cardiovascular risk [30].

### 3.1. Alternative Parameters Indicative of AS Severity

Dimensionless index, also known as Doppler velocity index (DVI), is an alternative parameter helpful to reduce the source of error related to LVOT cross-sectional area measurement, useful when image quality is inadequate. DVI is obtained by the ratio of *VTI _LVOT_* (on PW Doppler) *and VTI _AV_* on CW Doppler. Otherwise, it can be calculated as the ratio of the peak LVOT velocity to the peak AV velocity. It gives the size of the valvular effective orifice area as a proportion of the LVOT-CSA. DVI less than 0.25 indicates severe AS (Table 1) [31].

Ejection dynamic parameters, such as acceleration time (AT) and the ratio of AT to ejection time (ET), can discriminate patients with different stages of AS: an AT > 94 ms or an AT/ET ≥ 0.35 identifies severe AS with good accuracy [32].

In the presence of AS, the LV faces a double afterload: the valvular load due to AS and the arterial load, due to reduced arterial compliance. The valvulo-arterial impedance (Zva) is a useful echocardiographic parameter providing an estimate of the global LV hemodynamic load. It is obtained by the ratio of the estimated LV systolic pressure (the sum of systolic arterial pressure and mean pressure gradient) to the stroke volume indexed for the body surface area (Table 1). This parameter has a prognostic significance, thus guiding risk stratification and therapeutic choices: Lancellotti et al. found that high Zva (≥5 mmHg/mL/m^2^) was a powerful predictor of decreased cardiac event-free survival among asymptomatic patients with moderate to severe AS [33]. Hachicha et al. found a graded relationship between increased Zva and reduced overall survival in a population of asymptomatic AS patients [34].

### 3.2. Role of 3D Echocardiography in the Evaluation of Aortic Stenosis

**Figure 4:**
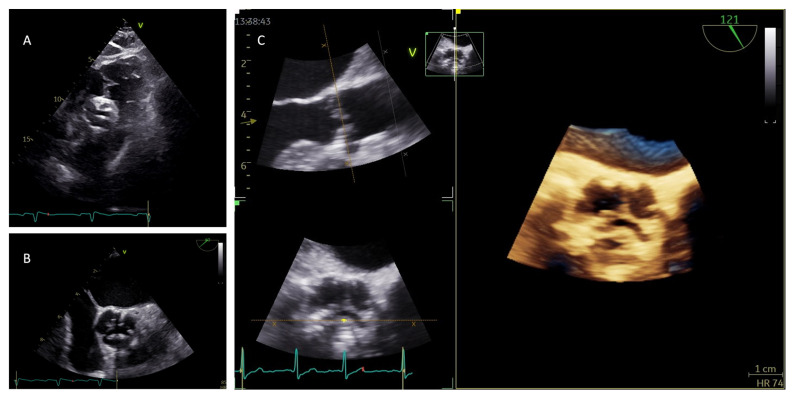
Evaluation of aortic valve morphology. (**A**) Subcostal view with 2D transthoracic echocardiography might provide a valid alternative in the absence of an adequate parasternal short axis view. (**B**) Short axis view with 2D transesophageal echocardiography. (**C**) 3D transesophageal echocardiography for the identification of the smallest aortic valve area.

The continuity equation is based on the assumption that the LVOT is circular, and the parasternal long-axis plane bisects the LVOT. However, the latter often presents a more elliptical shape, representing one of the main limitations of this calculation: in the case of an elliptical shape, the utilization of the antero-posterior diameter, which is generally smaller than the medio-lateral diameter, may result in underestimation of LVOT area and, thus, of stroke volume and effective AVA (6). In this context, the use of three-dimensional (3D) transesophageal echocardiography (TEE) is of fundamental importance and could overcome this limitation: 3D echocardiography indeed enables the measurement of the LVOT medio-lateral diameter and of the LVOT area planimetry. 3D TEE proved to be superior to 2D in the measurement of aortic annulus size and shape, providing results similar to MSCT or cardiac magnetic resonance (CMR)-derived diameters [35]. Ng et al. demonstrated that the use of 3D TEE planimetered annular area allowed the reclassification of 25% of patients from severe to moderate AS [36]. Moreover, in the absence of significant mitral regurgitation, by calculating 3D LV end-diastolic and end-systolic volumes, it is possible to obtain 3D-derived stroke volume to be used in the continuity equation [37]. In addition, the use of an echocardiographic contrast agent has proved to modestly improve the reproducibility of LVOT diameter measurement, so its use should be considered in patients with poor baseline image quality or poor interobserver reproducibility [38]. 3D echocardiography plays also an important role in the identification of the minimal orifice area when obtaining AVA with the planimetry method: it allows plane position control and any change in orientation in order to obtain the smallest AV orifice (Figure 4).

Furthermore, the use of 3D is paramount in the preprocedural planning of patients undergoing transcatheter aortic valve replacement (TAVR): besides the definition of aortic annular size, perimeter, and area, it also allows the measurement of the coronary ostium height.

## 4. Discordant Grading of Aortic Stenosis

The parameters above mentioned allow the description of different grades of aortic stenosis [39,40]:

- a valve area >1.5 cm^2^, a peak velocity between 2.6 and 2.9 m/s, a mean gradient <20 mmHg define a mild stenosis;
- a valve area between 1.5 and 1 cm^2^, a peak velocity between 3 and 4 m/s, or a mean gradient between 20 and 40 mmHg define a moderate stenosis;
- a peak velocity ≥ 4 m/s, a mean gradient ≥ 40 mmHg and an AVA ≤ 1 cm^2^, or an indexed (to body surface area) AVA (AVAi) ≤ 0.6 cm^2^/m^2^ are the criteria proposed by current guidelines to identify severe stenosis.

**Figure 5:**
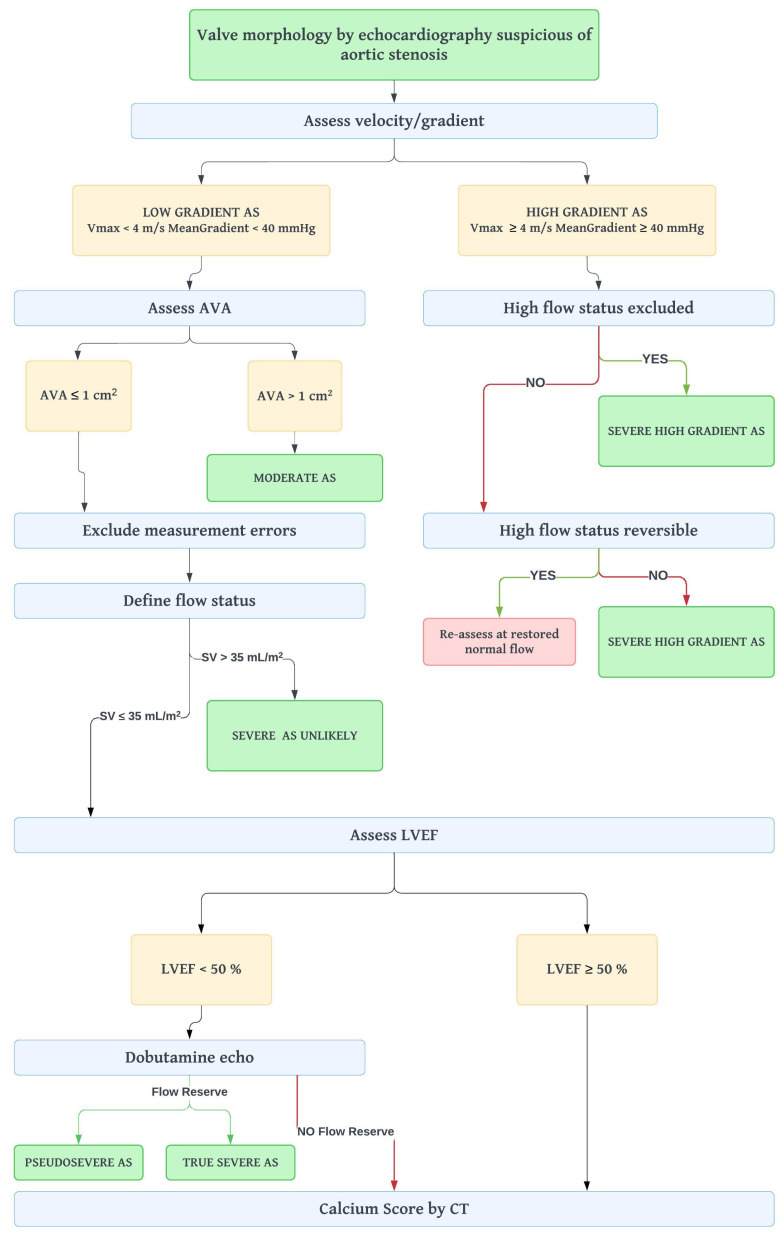
Stepwise approach to assess aortic stenosis severity. AS = aortic stenosis; AVA = aortic valve area; CT = computed tomography; LVEF = left ventricle ejection fraction; SVi = stroke volume index; V_max_ = maximum velocity.

Nonetheless, these criteria are not always consistent and the proportion of patients with discordant parameters is not negligible [41]. In the presence of a high gradient despite a value of AVA ≥ 1 cm^2^, concomitant hyperthyroidism, anemia, and other conditions determining transient high flow status should be excluded, as well as the presence of significant aortic regurgitation that might overestimate aortic mean gradient. Otherwise, the opposite case can be observed: valve area < 1 cm^2^, peak velocity < 4 m/s, and a mean gradient < 40 mmHg. As AVA calculation by continuity equation is a parameter prone to measurement error, firstly it is paramount to rule out measurement errors for LVOT area, LVOT and/or transaortic velocity. Nevertheless, a low gradient may be determined by severe hypertension, which compels to repeat the echocardiographic exam when blood pressure normalizes. Another important issue to consider is that, according to the Gorlin equation, an AVA < 1 cm^2^ better correlates with a mean gradient between 30 and 35 mmHg: this could explain the inconsistencies found in some of the patients analyzed. Almost 30–40% of patients with AVA ≤ 1 cm^2^ have a mean gradient < 40 mmHg; when the above-mentioned causes are excluded, an accurate evaluation of SV indexed (SVi) and LV ejection fraction (LVEF) is mandatory in order to discriminate between three forms of low-gradient severe AS (Figure 5) [42,43]:

- Classical low-flow low-gradient (cLFLG) AS, characterized by mean aortic transvalvular pressure gradient < 40 mmHg, SVi ≤ 35 mL/m^2^ and LVEF < 50%, and is detected in 5–10% of the AS cases;
- Paradoxical low-flow low-gradient (pLFLG) AS, characterized by mean aortic transvalvular pressure gradient < 40 mmHg, SVi ≤ 35 mL/m^2^ and LVEF ≥ 50%. In the vast majority, this type of AS affects women with small and concentric remodeled ventricles, that are responsible for the low flow state. In these patients, even the concomitant presence of mitral regurgitation, mitral stenosis, tricuspid regurgitation, and atrial fibrillation might explain the SV reduction.
- normal-flow low-gradient (NFLG) AS, instead, is defined by mean aortic transvalvular pressure gradient < 40 mmHg, a SVi > 35 mL/m^2^, and LVEF ≥ 50%. The reduction of the gradient despite a normal SVi can depend on a low transvalvular flow rate, calculated as SVi/LV ejection time. If the latter is prolonged, such as during bradycardia or because of systemic hypertension, the flow rate and consequently, the mean gradient will be lower [44].

As regarding SVi, the arbitrary threshold of 35 mL/m^2^ is under current debate [45]. Recently, Guzzetti et al. have proposed a sex-specific threshold (<40 mL/m^2^ for men, <32 mL/m^2^ for women). Using these thresholds, unlike the one recommended in the guidelines, it emerged that pLFLG was independently associated with increased mortality in both women (adjusted HR 2.05; *p* < 0.01) and men (adjusted HR 1.54; *p* = 0.042), even following AVR [29,46]. 

Recently, Stassen et colleagues have shown that in patients with moderate AS, discordant grading of AS can be detected in about 40% of cases [47]. Similarly to low-gradient severe AS, they identified three groups of low-gradient moderate AS: classical and paradoxical LFLG moderate AS and NFLG AS. Interestingly, both cLFLG and pLFLG moderate were independently associated with all-cause mortality at five-year follow-up, suggesting the need to better assess the phenotype of patients with discordant moderate AS in order to identify patients that would benefit from a closer follow-up [47]. 

### 4.1. Role of Dobutamine Stress Echocardiography 

In the presence of LFLG severe AS, dobutamine stress-echocardiography (DSE) is crucial in order to distinguish between *true severe* AS and what is called *pseudo-severe* AS [42,48].

As a matter of fact, an effective orifice area ≤ 1 cm^2^ and an LVEF < 50% may depend either on a truly stenotic valve or on LV dysfunction: prior myocardial infarction, for example, can cause the inability of the LV to completely open aortic valve cusps, despite the presence of a merely mildly or moderately stenotic valve (*pseudo-severe* AS).

When performing DSE, a low-dose dobutamine protocol is used: it involves the initial infusion of 2.5 or 5 mcg/kg/min of dobutamine and a dose increment every 3–5 min, up to a maximum dosage of 20 mcg/kg/min. AS velocity jet, mean gradient, AVA, and SV are measured at rest and at each stage of DSE, whereas LVOT diameter is measured at rest, and is used for calculating the SV. DSE aims to demonstrate how valve area, velocity, and gradient change as the flow rate grows. A truly severe AS is identified when AVA ≤ 1 cm^2^ at any flow rate, peak velocity ≥ 4 m/s, or a mean gradient ≥ 40 mmHg. Conversely, when the effective AVA increases >0.3 cm^2^ and the final AVA is >1 cm^2^, and the mean gradient remains <40 mmHg throughout DSE, the diagnosis is likely represented by pseudo-severe AS. Another factor to take into account is that the effective orifice area and gradient are strongly flow-dependent; nonetheless, transvalvular flow during DSE can result in differences between the patients due to several reasons, most commonly represented by the use of beta-blockers [44,49]. Hence, there is the need for a new parameter, projected AVA (AVAproj) at a normal flow rate (Table 1), which proved to overcome this limitation and to make DSE more reliable in detecting truly severe aortic stenosis [50,51,52]. Transvalvular flow rate is obtained by dividing SVi by LV ejection time (LVET); a flow rate of 250 mL/s (the median value of the normal flow range) is chosen as the standardized flow rate for every patient to calculate AVAproj as follows:

AVAproj = AVArest + VC × (250 − Qrest)

A simplified method makes the calculation of AVAproj easier:

VCsimpl = (AVApeak − AVArest)/(Qpeak − Qrest)

VC is the valve compliance, AVArest and Qrest are the aortic valve area and the mean transvalvular flow at rest, AVApeak and Qpeak are the aortic valve area and the mean transvalvular flow during peak DSE. 

Clavel et al. demonstrated that AVAproj ≤ 1 cm^2^ (calculated with the conventional or simplified method) defines severe stenosis even better than other measurements during DSE [51]. While DSE plays a key role in LFLG AS, its role in paradoxical LFLG AS is under investigation. Despite the preserved LVEF, transvalvular flow throughout systole is below normal, thus it is crucial to detect true severe AS. PLFLG often affects patients with hypertrophied ventricles, a condition that, together with preserved EF, could invalidate the use of DSE. On the contrary, Clavel et al. proved the accuracy of DSE in distinguishing between different forms of AS, even in PLFLG [53]. 

The main purpose of DSE is patient therapeutic management: patients with pseudo-severe AS may not benefit from aortic valve replacement (AVR) and heart failure therapy should be considered in these patients. On the other hand, patients with truly severe AS may benefit from intervention: they may experience improvement in terms of LVEF when treated with AVR since LVEF reduction actually depends on the small effective orifice area of a severely calcified and stenotic valve. In this group of patients, DSE helps to identify the absence of flow reserve (the lack of increase in stroke volume ≥20% from baseline), which is a condition associated with high operative mortality and poor long-term prognosis [54,55]. Conversely, Sato et al. demonstrated that AVR improves survival regardless of the presence of flow reserve. Thus, they suggested that there is no more statistical association between flow reserve and risk stratification [56]. 

### 4.2. Role of Exercise Stress Echocardiography

Symptomatic severe AS represents a class I indication for AVR, with no need for further evaluation [25]; exercise testing is contraindicated in this population. However, patients affected by severe AS often limit their daily activities to avoid symptoms occurrence, such as dyspnea, thus they can be mistakenly classified as asymptomatic. Therefore, exercise stress echocardiography (ESE) is recommended to unmask symptoms or abnormal blood pressure responses in this population, in order to obtain a more punctual risk stratification. Approximately one-third of patients exhibit exercise-limiting symptoms during exercise tests and worse outcomes at follow-up [57]. When using a treadmill, images should be acquired at baseline and immediately post-exercise, while they should be recorded at low and peak workloads if a supine bicycle exercise is used [58]. Peak aortic velocity and mean gradient, LVEF, and trans-tricuspid pressure gradient for pulmonary artery systolic pressure (PASP) estimation are the minimum echocardiographic parameters to be acquired. 

An increase in mean aortic pressure gradient by ≥18–20 mmHg, the absence or limitation of LV contractile reserve (decrease or no change in LVEF) and induced pulmonary hypertension (PASP > 60 mmHg during exercise) are markers of poor prognosis [58].

ESE has an impact on therapeutic management since demonstrable symptoms or sustained falls in blood pressure (>20 mmHg) during exercise testing have been retained as an indication for intervention in asymptomatic patients with severe AS, in the latest European Society of Cardiology (ESC) guidelines (class I and class IIa, respectively) [25]. 

## 5. Aortic Stenosis-Related Structural and Functional Changes

Cardiac damage in AS is not limited to the aortic valve but is a disease affecting the entire cardiovascular system that can be characterized by significant alteration of the other cardiac chambers. Severe AS is associated with chronic pressure overload that causes an increase in LV wall stress and leads to a hypertrophic response of the cardiomyocytes. Although effective in an early stage, this compensatory effect increases myocardial oxygen demand, in the absence of a balanced increase in the coronary capillary network. This will progressively lead to myocyte degeneration, death, and fibrosis [59,60]. When wall stress exceeds the compensating mechanism, LV contractile function declines [61]. Moreover, the increased left atrial pressure is retrogradely transmitted through the pulmonary vasculature, leading to right heart remodeling and dysfunction [62,63]. In AS patients, echocardiographic markers of pulmonary hypertension (PH) and right ventricle (RV) systolic dysfunction have a prevalence of about 30 and 25%, respectively [64,65,66]. For this reason, in the presence of AS, a comprehensive echocardiographic evaluation should take into account all the related anatomical and functional changes occurring in the other cardiac structures. Généreux et al. almost recently proposed a new staging classification characterizing the extent of extra valvular cardiac damage among patients with severe AS. Patients were categorized into five independent and not additive stages, depending on the echocardiographic evidence of myocardial structural changes, haemodynamic parameters, and indices of LV and RV dysfunction. Interestingly, the stage of cardiac damage was shown to be one of the strongest predictors of 1-year death: for each stage increment, one year mortality risk increased by about 45% [67]. Moreover, Avvedimento et al. demonstrated that the extent of cardiac damage at baseline significantly affected the risk of mortality at one year after transcatheter aortic valve implantation [68]. 

A detailed detection of cardiac damage should also include an evaluation of global longitudinal strain (GLS) by 2D speckle-tracking strain imaging, one of the most powerful tools to evaluate subclinical LV dysfunction. Actually, the longitudinal function is largely governed by the subendocardial fibers, which are first affected by the reduced myocardial perfusion and by the pathological changes (hypertrophy, increased wall stress, and reduced arterial compliance) associated with AS. This makes decreased GLS the earliest alteration seen in the presence of AS [69]. In addition, GLS has been proven to be superior to LVEF in predicting survival outcomes [70,71,72,73,74]. Magne et al., in a recent participant data meta-analysis of ten studies, including 1067 asymptomatic patients with significant AS and LVEF > 50%, demonstrated the strong relationship between LV-GLS and all-cause mortality with a >2.5-fold increased risk of death in patients with impaired LV-GLS. The Authors identified a value of 14.7% as the optimal cut-off to identify patients at higher risk of death [75]. However, most of the studies included in this meta-analysis involved a single-vendor speckle-tracking strain software, and significant inter-vendor variability could limit a large-scale clinical use of GLS. For this reason, Thellier et al. tested the prognostic significance of an absolute value of GLS ≤ 15%, obtained with vendor-independent speckle tracking strain software, in a population of severe AS with preserved LVEF and no or mild symptoms, showing a 2-fold increased risk of mortality in those with reduced GLS [76]. These results support the use of GLS as an additive parameter for clinical decision-making, helping to identify patients who would more likely benefit from earlier intervention. Also, the evaluation of RV systolic function through GLS has provided prognostic implication, with a value of RV longitudinal strain < 13% at rest related to worse 2-year survival, and <14% post stress (measured during low-dose DSE) even stronger predictor of mortality [77]. 

Despite its recognized diagnostic and prognostic value, GLS main limitation is its load dependence, which could be relevant in patients with severe AS. Recently, non-invasively derived LV myocardial work, being less load dependent, demonstrated to overcome GLS limitation [78] and provide a more accurate evaluation of LV performance [78,79]. In AS patients, in order to calculate myocardial work indices, it has been suggested to estimate LV pressure by adding the mean aortic transvalvular gradient to the aortic systolic pressure measured at the brachial artery with a cuff manometer [80,81]. Fortuni et al. proved that LV global constructive work (GCW) and global work index (GWI) had significantly lower values in LFLG AS patients with New York Heart Association (NYHA) functional class III or IV heart failure [81]. Furthermore, when asymptomatic moderate to severe AS patients were stratified according to the stages of cardiac damage, GWI appeared to be significantly reduced in the advanced stages, and values of GWI ≤ 1951 mmHg% or GCW ≤ 2475 mmHg% were independent predictors of all-cause mortality and cardiovascular death at 4-year follow-up [82]. 

## 6. Artificial Intelligence in Aortic Stenosis Severity Assessment

As previously described, diagnosis of severe AS could be very challenging and not always straightforward. In this context, the use of artificial intelligence (AI) algorithms, through the analysis of some echocardiographic features, is demonstrated to provide valuable support in the diagnosis of AS. In particular, AI (as a Diagnostic Precision Algorithm) has been used to help cardiologists untangle cases where AS severity was not clear for incongruence of echocardiographic severity parameters (jet velocity, AVA, and mean gradient), defining a likelihood of severe AS with only a 2.1% average error, thus reducing the rate of misdiagnosis, shortening treatment delays and improving patient outcomes with curtailment of the cost burden on the healthcare system arising from delayed care. Interestingly, Playford et al., have trained AI to impute AVA from other echocardiographic data without the need for any LVOT measurements, which are notoriously an important source of error in AVA estimation through the continuity equation. [3,83,84] Conversely, Thalappillil et al. showed a good correlation between aortic annulus measurements made by an automated echocardiographic software with the ones obtained from a single radiologist analyzing MSCT images [85]. A further application of deep learning, and in detail of convolutional neural networks, that is emerging recently is to identify and eventually diagnose AS only with limited imaging data sets, like a single parasternal long axis acquisition. This could be helpful to make AS screening more accessible and to have an early diagnosis in asymptomatic patients [86,87]. A machine learning model has also proven to be useful in predicting the evolution of mild-to-moderate AS in severe valvular disease at one, two or three years follow-up, and thus in defining the timing of the next echocardiographic follow-up examination, avoiding up to 49% of unnecessary echocardiographic examinations per year [88]. 

Additionally, Lachmann et al. demonstrated that an artificial neural network could categorize patients through unsupervised agglomerate clustering into groups based on echocardiographic and right heart catheterization variables, which resulted very similar to the ones described by Genereux et al. In this study, it appeared that post-TAVR long-term mortality significantly differs between groups, independently from transvalvular gradients, and artificial neural network could accurately assign new patients, with high sensitivity and specificity, to these predefined clusters [89]. Comparable results have emerged in other studies, showing that, through the analysis of various echocardiographic features, AI algorithms could re-classify patients with mild to severe AS into risk categories that have a more robust association with outcomes, compared to standard AS severity classification [90]. 

Therefore, AI is progressively advancing in the cardiology scenario with a lot of pioneering applications. In the not-so-distant future, cardiologists could use AI to screen asymptomatic patients for AS, to better diagnose it without any uncertainty, and to deliver a more tailored therapy and follow-ups, optimizing the timing of AVR, avoiding useless exams, and ameliorating short- and long-term patients’ outcomes.

## 7. Limitations of Echocardiography in the Assessment of Aortic Stenosis

Echocardiography requires skilled operators to evaluate valve stenosis. The reliability of the assessment can vary depending on the operator’s experience and expertise. Proper optimization of Doppler settings and angle correction is, for example, crucial to measuring transvalvular velocities, pressure gradients, and effective orifice area. Moreover, limiting factors for echocardiogram accuracy are patient positioning and suboptimal acoustic windows, which can affect image quality. Echocardiography provides a 2D and 3D view of the aortic valve. However, due to the anatomy and positioning of the valve, complete visualization could be challenging. In particular, aortic calcification is very common in AS: calcification can create artifacts on the echocardiographic images, causing shadowing and obscuring the underlying structures. This can make it arduous to evaluate valve morphology and estimate the degree of stenosis. In complex cases, combining echocardiography with other imaging modalities such as CT or CMR can yield a more comprehensive evaluation. These methods provide detailed information about aortic valve anatomy and hemodynamics, calcification distribution, and evaluation of myocardial function and associated pathology. In particular, in the presence of discordant echocardiographic markers of AS, electrocardiogram-gated CT scans allow the measurement of aortic valve calcification with the use of the Agatston method: an aortic valve calcification score higher than 1300 AU in women or 2000 AU in men should be considered severe [91].

## 8. Conclusions

The echocardiographic diagnosis of AS should always be obtained by a standardized and systematic multiparametric approach. However, it still poses important challenges, thus entailing the risk of diagnostic delays with serious consequences on the patient’s outcomes and therefore, a multiparametric echocardiographic exam should be performed. In this context, TEE and 3D TEE are of fundamental importance in helping the echocardiographer to better define the anatomic AVA and the SV. A complete echocardiographic evaluation cannot be separated from the assessment of all the structural changes related to AS; in particular, the evaluation of LV function, GLS and MW proved to be important methods in the decision-making process, providing diagnostic and prognostic information. The recently developed AI algorithms are emerging as promising tools in assisting the diagnosis of AS, helping to improve the diagnostic accuracy.

## Figures and Tables

**Table 1:** Echocardiographic parameters for aortic stenosis severity assessment. AS = aortic stenosis; AV = aortic valve; AVA = aortic valve area; AVAproj = projected aortic valve area; AoA = cross-sectional area of the ascending aorta; BSA = body surface area; CSA = cross-sectional area; ELI = energy loss index; EOA = effective orifice area; LVOT = left ventricle outflow tract; N = number of instantaneous measurements; P = pressure; Q_rest_ = flow at rest; sBP = systolic blood pressure; SVi = stroke volume index; v = velocity; VC = valve compliance; VR = velocity ratio; VTI = velocity time integral.

	Units	Formula/Method	Severe AS Cutoff
AS jet velocity	m/s	Direct measure	>4.0
Mean pressure gradient	mmHg	ΔP = Σ4v2/N	>40
EOA	cm^2^	Continuity equationAVA = (CSALVOT ∗ VTILVOT)/VTIAV	<1.0
Indexed EOA	cm^2^/m^2^	EOA normalized by BSA	<0.6
Dimensionless index		VR = VLVOT/VAV	<0.25
Energy loss index	cm^2^/m^2^	Indexed EOA accounting for ascending aorta ELI = [(AVA ∗ AoA)/AoA−AVA]/BSA	<0.5–0.6
Valvuloarterial impedence	mmHg/mL/m^2^	ZVA = (sBP + ∆Pnet)/SVi	4.5–5
Projected valve area at normal flow	cm^2^	Estimated EOA at normal flowAVAproj = AVArest + VC ∗ (250−Qrest)	<1.0

## Data Availability

Data are taken from literature.
